# Psychometric properties of dyadic data from the Marital Quality Scale of Indonesian Javanese couples

**DOI:** 10.1186/s41155-023-00279-7

**Published:** 2023-11-27

**Authors:** Siti Rohmah Nurhayati, Farida Agus Setiawati, Rizki Nor Amelia, Lara Fridani

**Affiliations:** 1 Department of Psychology, Universitas Negeri Yogyakarta, Jl. Colombo No.1 Karangmalang, Sleman, Yogyakarta, 55281 Indonesia; 2 Department of Psychology, Universitas Negeri Yogyakarta, Jl. Colombo No. 1 Karangmalang, Sleman, Yogyakarta, 55281 Indonesia; 3 Department of Integrated Sciences, Universitas Negeri Semarang, Sekaran Kec Gunung Pati, Semarang, 50229 Indonesia; 4 Department of Early Childhood Education, Universitas Negeri Jakarta, Jl. Rawamangun Muka Raya No. 11, RT 11/Rw 14, Jakarta, Timur Indonesia

**Keywords:** Javanese marriage, Marital quality, Psychometric property, Relationship, Well-being

## Abstract

**Background:**

Researchers have demonstrated that various measurement concepts and dimensions depend on context and timing.

**Objectives:**

The current study aimed to determine the psychometric properties of the Javanese couples’ marital quality scale based on validity and reliability

**Methods:**

In total 840 participants or 420 marital dyad from Java, Indonesia, were involved in this study. The psychometrics properties scale was analyzed using exploratory and confirmatory factor analysis, convergent and discriminant analysis, and composite reliability.

**Results:**

The exploratory factor analysis found relationship quality to consist of support, physical proximity, warmth, communication, acceptance and respect, role sharing, and responsibility factors. Well-being quality consists of happiness, harmony, and problem-solving. The fit of the measurement model was obtained using confirmatory factor analysis. The fit model was also found in the husband’s and wife’s groups, with no differences between them. The high correlations between wife-husband factors also proved the validity based on convergent and discriminant evidence. The reliability coefficient was high for each dimension and construct.

**Discussion:**

This analysis shows that the marital quality scale developed has information on psychometric properties that can be useful for researchers and the practicians using the marital quality instrument of Javanese couples in particular.

## Introduction

Considering the global increase in divorce rates, the study of marital quality has attracted widespread attention as an important research area (Razak et al., 2015). In Indonesia, divorce rates increased by 5% during the COVID-19 pandemic, with this increase mostly ascribed to economic difficulties (Fauziah et al., 2020). Family disharmony also played a significant role due to an imbalance between solitary activities and time spent together, domestic violence, changes in communication patterns, and the age factor in fostering a household. In Indonesia, the majority of divorces are filed by couples who are already married between 31 and 40 years old (Yumarni & Suhartini, 2019) and have been married for less than 5 years (Kendhawati & Purba, 2019). The cause, generally, is marital conflict (Apriliani & Nurwanti, 2020; Thomas & Maria, 2021). Many divorces occur in the early stages of marriage due to the couple’s failure to develop marital quality.

Marital quality is an individual’s subjective, global assessment of their relationship and behavior (Fincham & Bradbury, 1987); good marital quality is one way to prevent divorce. The high divorce rate indicates that many married couples have not been able to develop good marital quality (Setiawati & Nurhayati, 2020), even though divorce is a major stressor for everyone involved, with negative consequences for the mental and physical health of all family members (Damota, 2019). A meta-analysis found that good marital quality will also have a positive impact on health (Carr et al., 2014). Moreover, they found marital quality to be very closely related to self-evaluations of one’s whole life (reflected in judgments of life satisfaction) and moment-to-moment experiences of happiness while performing daily activities.

Marital quality can be measured in various ways, such as self-report measures, which are most commonly used by researchers (Delatorre & Wagner, 2020). The other way is by recording a video of a couple discussing their relationship’s problems, then coding the recording to identify behaviors indicating high marital quality or supportive behavior and behaviors indicating low marital quality, such as hostile or withdrawn behavior (Thomas & Fletcher, 2003). Regardless of the method, the measurement of marital quality generally has either clinical objectives used to identify marital problems, or basic research objectives used to test theories related to the function and associated behaviors of marriage (Johnson, 1995).

Some theories in the field of psychology are related to culture, loaded with values, and consider the characteristics of a particular region (Bretherton, 2015). Therefore, psychological phenomena must be understood in their ecological, historical, philosophical, religious, and cultural contexts (Broman, 2005; Bulanda & Brown, 2007; Kim et al., 2006; Riveros Munévar et al., 2021). In this context, marital quality studies align with the theory of psychology, which is based on indigenous culture and indigenous realities. In other words, indigenous psychology is relevant to marital quality, considering that research has demonstrated that the dimensions of marital quality can vary according to the context in which the research is conducted (Allendorf & Ghimire, 2013; Hassebrauck & Fehr, 2002; Verhofstadt et al., 2006; Xiong et al., 2016). The marital quality dimension is not universal, but it is affected by context and time. Furthermore, marital quality has been measured as part of longitudinal and cross-sectional studies (Aggarwal et al., 2017; Brauner-Otto et al., 2020; James, 2015; Johnson, 1995; Kronmüller et al., 2011; Liu et al., 2021; Lo et al., 2017; Rohrbaugh et al., 2006; Stokes, 2017; Tracy & Utz, 2020).

In the current study, we determined marital quality by drawing on the Indonesian concept of “quality” or “the level of good and bad things.” Quality of marriage was defined as good or bad based on certain criteria (Departemen Pendidikan Nasional, 2012). In Indonesia, the concept of marriage extends beyond the relationship between a man and woman and encompasses the realms of religion and culture. According to s. 1 of the Marriage Law 1974 (UU), the goal of marriage is to build a happy and eternal household based on the one and only God. For this reason, marriage requirements reflect religion-based rules which are recorded by the Ministry of Religion. Further, local culture may affect marriage customs. For example, the Javanese Religious wedding ceremony is an important element of marriage as it serves as a foundation for a sincere, loving relationship between women and men (Roibin, 2015). Likewise, Javanese religious and cultural teachings regulate husband–wife relationships, relationships with children, and relationships between family members and the community (Sumarto., 2020). Thus, in Java, religion and culture play an important role in establishing the values needed for a good quality marriage. The current study focuses on local conceptualizations of a good marriage and recognizes that the concept of a happy family is not located at an individual level but is conceptualized within the Indonesian social system.

In this study, Javanese culture was chosen, as its people form the largest ethnic group in Indonesia and have a relatively well-established history and unique marital characteristics. As a Southeast Asian indigenous group, most Javanese people still sanctify marriage as a holy event that should be treated with respect. Furthermore, once a couple marries, the wife’s role is seen as fulfilling her husband’s demands rather than having demands of her own (Wismanto, 2011). This kind of belief is based on a patriarchal system that treats a wife as a *kanca wingking,* or “friend from behind”: Javanese marriage customs positions women behind, or at a lower position, than their husbands (Handayani & Novianto, 2004; Magnis-Suseno, 2001; Putri & Lestari, 2015). *Kanca wingking* also means that women must always encourage and support their husbands. This belief is related to Javanese culture, which commands women to obey their husbands, as illustrated in the saying *suwarga nunut neraka katut*, or “follow to heaven also to hell.” If a husband goes to heaven, his wife will also go to heaven; however, if a husband goes to hell, even though his wife has the right to enter heaven because of her good deeds, she will not go to heaven because she has to follow her husband wherever he goes (Putri & Lestari, 2015). Javanese culture also places honorific value in treating the wife as *sigaraning nyowo,* or “half of the soul”—her husband’s soulmate in life (Mardiana, 2017). These various terms indicate that marriage has many values in Javanese culture that must be implemented according to their respective roles.

The concept that seems to discriminate the women is only evolved in the public area as something that is ideal for Javanese culture. However, in social reality, it is not necessarily in accordance with the ideal picture of a husband and wife relationship (Magnis-Suseno, 2001). In real-life practice, it needs to be determined by the situation or “*ndelok kahanan”* (Handayani & Novianto, 2004). The existence of a cultural shift also affects husband and wife relations at this time to become more egalitarian (Nurhayati, 2017). In the past, the husband, as the head of the family, contributed most to making a decision, but now it has shifted to a joint decision of husband and wife (Murniatmo et al., 1996).

Sunarti et al. (2005) have conducted research about marital quality in Indonesia using Conger and Glen’s theory (Keating et al., 1995). The construct of the instrument consists of dimensions such as marital satisfaction and happiness. The indicators include many factors such as commitment, trust, marital value, communication, family togetherness, equality, relationship with extended family, expression of affection, love, sexual relations, equality of interest, economy, and family income. The Enrich Marital Satisfaction Scale (Fowers & Olson, 1993) has also been modified for use in Indonesia (Istiqomah & Mukhlis., 2015). Other scholars (Soraiya et al., 2016) have used Hazan and Shaver (1987) to measure marital relations based on attachment behavior, which was divided into security, avoidance, and anxiety dimensions.

Previous studies on marital quality have been conducted with a sample of Javanese people. However, research on Javanese people’s marital quality, which is based on Javanese culture, is relatively limited, particularly regarding marital quality instruments. The construct of marital quality has been studied by involving people from one of the regions in Java, Yogyakarta (Nurhayati, 2017; Nurhayati & Helmi, 2019). The concept was built using mixed-method research with a sequential exploratory design that distinguished marital quality from the dimensions of marital relationship quality and marital well-being quality. These concepts were explored qualitatively using an open-ended questionnaire and tested in a measurement model that resulted in several separate marital quality indicators in two dimensions. The quality relation included the interpersonal processes demonstrating the existence of connectedness between husbands and wives, manifested through support, attachment, cooperation, communication, warmth, and acceptance between husbands and wives. Well-being quality emphasizes the intrapersonal connection between spouses, based on the indicators of happiness/peace, harmony, and problem-solving. The current study refers to Nurhayati (2017) but focuses on a broader research subject, that is Javanese culture.

Differences in the subject of the measurement sample of instruments have resulted in the development of different constructs and psychometric properties. The fundamental psychometric properties in assessing the standard of measurement from the American Educational Research Association (AERA), American Psychological Association (APA), and National Council on Measurement in Education (NCME), are validity and reliability (Dardick & Mislevy, 2016; Fietzer & Ponterotto, 2015; Furr & Bacharach, 2014; Lovler & Miller, 2016). The validity of the instrument must be supported by evidence from empirical research, and evidence for the validity measure instrument can be evaluated based on the internal structure and relations with other variables.

A study using the Kansas Marital Satisfaction Marital Scale demonstrated evident validity from a confirmatory factor analysis (CFA) and the reliability from internal consistency, inter-item correlations, and corrected item-total correlations (Omani-Samani et al., 2018). In another study, the construct of marital quality in Ghana resulted from a factor loading of 0.5, also as the coefficient reliability (Miller & Kannae, 1999). Additionally, a study on the marital quality scale from 150 Agartala women who have been married demonstrated the reliability of internal consistency 0.91 and test-retest reliability 0.83 (Bhattacharjee & Banik, 2016). The validity of the Chinese marital quality scale has also been examined using exploratory and confirmatory factor analyses, discriminant validity, and reliability (Huiping Zhang et al., 2013). Marital quality in urban China has been examined using 15 positive marital quality (PMQ) items and five negative marital quality (NMQ) items or marital instability and demonstrated the reliability of the PMQ 0.93 and NMQ 0.83 (Huiping Zhang, 2015). In Germany, a property psychometric study involving 1431 respondents used an exploratory and fit model of a confirmatory factor analysis as evidence of the instrument’s validity. The coefficient of Cronbach’s alpha was 0.94 demonstrating high reliability (Zimmermann et al., 2019).

In existing studies, the concept of marital quality tends to adapt to existing research, even though the dimensions of marital quality may vary due to different contexts in which the research is conducted, and several psychometric properties are evidence. Therefore, a clear understanding of the marital quality of Javanese people in Indonesia, a large region dominated mainly by Javanese people, is essential. Based on the above discussion, this study aimed to determine the psychometric properties of Javanese marital quality. The psychometric properties focused on the evidence of validity based on internal structure, convergent, and discriminant analysis. Reliability estimates were also measured in this study to determine how the internal consistency of the measurement results score the marital quality instrument was produced.

## Methods

### Participants

The data source or population of this study was taken from married Javanese people living in the Special Region of Yogyakarta, Solo, Banyumas, and Pekalongan. The four regions have fulfilled several criteria as being representative of Javanese people as they are the central area of Javanese culture (Yogyakarta and Solo), mountainous regions (Banyumas), or coastal areas (Pekalongan). Eight hundred forty Javanese participants (420 marital dyads) were involved in this research. The average age of the participants was 42 years in study one and 44 years in study 2, and they have been married, on average, for 17 and 19 years. Most participants had a secondary school background education level, followed by primary and graduate background education. Most participants identified themselves as Javanese in high and moderate. Table 1 presents the demographic data of the participants. The research sample selection was determined using a multi-stage random sampling technique. The first stage was to perform randomization based on the clusters or sub-districts, and the second stage was to perform randomization on a village or urban village.

**Table 1:** Demographic of the participants

Demographic	Study 1				Study 2			
	Min	Max	Mean	SD	Min	Max	Mean	SD
Age	20	69	42	10.46	19	75	44	11
Marital age	1	60	17	0.16	1	60	19	11.55
Number of children	0	7	2	1.15	0	7	2	1.25
Sex (frequency)								
Male	214 (53.5%)				205 (47%)			
Female	186 (46.5%)				235 (53%)			
Educational level n (frequency)								
No school	1 (0.25%)				4 (0.91%)			
Primary	87 (21,75%)				105 (23.86%)			
Secondary	254 (63.5%)				290 (65.91%)			
Graduate	58 (16.5%)				41 (9.32 %)			
Javanese identification (frequency)								
Low	33 (8.3%)				66 (15%)			
Middle	195 (48.8%)				140 (31.8%)			
High	172 (43.5%)				234 (53.2%)			

### Instruments

The marital quality scale consists of 57 items divided into 39 items of relation quality and 18 items of well-being. The specification of the study instruments is written in Table 2. For example, the items representing *communication with each other*, “I and my partner used to exchange experiences.” The item represents acceptance, “I and my partner need each other.” and the item represents *problem-solving*, “When there is a family problem, we solve it together.” This scale has adequate validity based on the content, proven by Aiken’s score moving between 0.82 and 0.97, with a mean for all items of 0.91. The result from trials on 256 Yogyakarta citizens obtained that the power on relation dimensional items is shifting between 0.228 and 0.635, with a mean of 0.456. Meanwhile, on well-being, dimensional items are turning between 0.475 and 0.744, with a mean of 0.657. The construct reliability has already met the standard of 0.824 for the relation construct, and 0.802 for the well-being construct.

**Table 2:** Measuring instrument specifications

Components	Aspects	Indicator	No items
Relationship quality	Attachment and warmth	Communication between husband and wife	1–3
		Mutual understanding between husband and wife	4–6
		Mutual trust between husband and wife	7–9
		Mutual love between husband and wife	10–12
		Complementing each other	13–15
		Maintaining togetherness	16–18
		Maintaining sexual life	19–21
	Respect	Mutual respect between husband and wife	22–24
		Reciprocal support between husband and wife	25–27
		Mutual acceptance between husband and wife	28–30
	Cooperation	Role sharing in domestic matters	31–33
		Carrying out respective roles with responsibility	34–36
		Solving the family problems together	37–39
Well-being quality	Harmony	Minimum conflict	40–42
		Solving the family problems	43–45
	Peace	Peaceful feeling	46–48
		Comfortable feeling	49–52
	Happiness	Feeling grateful of the marriage	52–54
		Feeling happy of the marriage	55–57

The instrument uses a Likert scale, and the research subjects were asked to respond to the statement items on a scale of 1 to 5. The scores were 1 = very inappropriate, 2 = inappropriate, 3 = neutral, 4 = appropriate, and 5 = very appropriate. For example, in the statement item, “My partner and I care about each other,” if the participant responded with “very inappropriate,” this suggests that they (the husband and wife) do not care about each other. Therefore, the participant was assigned a score of 1. If the participant’s response was “very appropriate,” their response indicated that the couple cared for each other, and the response was assigned a score of 5.

### Procedure

This study used a quantitative method to observe the psychometric properties of Javanese people through a marital quality instrument. The data were collected using the marital quality scale created by Nurhayati (2017), based on the Yogyakarta people. The researchers have been permitted to use this instrument to measure marital quality on a larger scale, namely Javanese people. Before being asked to respond to the measurement tools, the respondents were offered their willingness to be research respondents by signing an informed consent sheet and stating that they were willing to complete the instruments honestly, as experienced in their households. The data were collected only for research purposes, and participants’ identities remained confidential. Participants had the option of providing their own pseudonyms.

### Statistical analysis plan

The psychometric properties of Javanese people in the instrument were analyzed based on validity and reliability. The evidence of support validity was studied using factor analysis, convergent, and discriminant. An exploratory factor analysis (EFA) and confirmatory factor analysis (CFA) were performed to construct the measurement. The EFA was performed to reduce the data and determine the number of factors that underlined the measuring instrument. The interpretation of the EFA results began by examining the correlation matrix. Tabachnick and Fidell (2001) recommend that good items have a minimum correlation coefficient of 0.3. The analysis was continued by examining the sampling adequacy through the Kaiser-Meyer-Olkin Measure of Sampling Adequacy (KMO-MSA). Generally, KMO values above 0.5 indicate sufficient samples. Bartlett’s test of sphericity was also used to determine whether the correlation matrix was not an identity matrix if it was significant (sig < .05), and the factor analysis could be continued.

Data extraction was used to simplify the number of variables or items based on the similarity of the underlying properties. The extraction method can be in the form of non-iterative estimation (principal component method, principal factor method, image analysis, and Harris’ canonical factor analysis) and iteration (maximal likelihood, unweighted least squares, iterative principal component, and alpha factor analysis). Both models can be analyzed using a large sample. In turn, this research used the principal component method. The number of factors from EFA based on an eigenvalue is more than 1, and each factor has more than three items (Hair et al., 2019). Factor rotation was used to obtain the factor loading pattern to achieve a better interpretation. The final step was to determine which items were included in which factors and, at the same time, name new factors based on the grouping of items. For loading items into factors, Plucker (2003) demonstrated that many researchers had used a factor loading limit of 0.30; however, in the end, researchers need to consider the convenience factor when determining the cut-off for the interpretation. In this study, item loading into the factor was based on a loading factor of more than 0.3, the largest loading factor, and the suitability of the content items to put it into one dimension.

The factors formed from the results of the EFA were then analyzed using a CFA. It can be used when researchers have theoretical or empirical knowledge about the structure of the underlying latent variables (Schivinski, 2013). The CFA was carried out using the Jamovi program. The results of the analysis were obtained by examining the data analyzed with the fit of the theoretical construct based on the index of the fit model. There are various fit index models as reported by the program, including root means square error of approximation (RMSEA), standardized root mean square residual (SRMR), Tucker-Lewis index (TLI), Bentler-Bonett Normed Fit Index (NFI), and Comparative Fit Index (CFI) (Hu & Bentler, 1999; Montoya & Edwards, 2021).

The confirmatory factor analysis multi-group on man or husband and woman or wife has been conducted on a latent variable. The Latent was obtained from the previous EFA Analysis. The invariance models of the multi-group were reported from the index of the fit model. The differences between the two models could be calculated from the difference in chi-square and CFI. The chi-square value of the model difference, which is lower than the chi-square table by 5%, indicates that Ho is accepted. There is an invariance model between groups. The CFI comparison value between the two models that are above 0.01 indicates Ho rejected. There is a difference in the value of the model fit (Cheung & Rensvold, 2002). The baseline, unconstrained, and constraints model fit were calculated to determine the differences between groups. Several constraint parameters are loading factor, threshold, mean, and residue.

In this study, the reliability estimation of instruments was carried out by analyzing Alpha Cronbach and McDonald’s Omega (McDonald, 1999). The reliability is a coefficient of reliability based on the analysis of the variability in each dimension. Coefficient alpha was conducted to estimate the reliability of each dimension. The reliability estimates the reliability of composite scores using structural equation modeling (SEM) for non-homogeneous components (Widhiarso & Ravand, 2014). As it is developed under the SEM framework, the construct reliability coefficient can be trusted when applied to large samples (Raykov, 1997).

## Results

### Construct measurement

The data of this research was carried out simultaneously with as many as 840 respondents (420 pairs of husband and wife). EFA analysis was carried out with 400 respondents who were taken randomly, and the remaining 440 were used for CFA analysis. The EFA were analyzed to explore the construct of Javanese people’s marital quality. The instrument consisted of 39 items on the dimension of Relations Quality and 18 items on the dimension of well-being quality. All items had a correlation coefficient and eigenvalue above 0.3. Based on these criteria, the items were interrelated and could be used in the data reduction of factor analysis. The analysis was continued by examining sampling adequacy through the KMO-MSA. The KMO-MSA values for the dimensions of relationship quality and well-being quality were 0.939 and 0.929, respectively. The analysis demonstrated the significance of Bartlett’s test of sphericity < 0.00 for each dimension tested. Accordingly, these requirements were met because the significance level was below 0.05. Because the KMO-MSA and Bartlett’s test of sphericity requirements were fulfilled, the factor analysis could be continued.

The next step was to determine the number of factors. Based on these eigenvalues, the dimension of relationship quality was composed of seven factors with a total contribution (cumulative variance) of 61.839%, while the dimension of well-being quality was composed of three factors with a total contribution of 65.78%. A summary of the results of exploratory factor analysis for each dimension is presented in Tables 3 and 4. Furthermore, each factor was named according to the meaning of its constituent items and also was adjusted to the specifications of the instrument as shown in the tables.

**Table 3:** The factors and items of the dimension relations

No item	Factor loading						
	F1	F2	F3	F4	F5	F6	F7
1				0.553			
2				0.591			
3				0.673			
4				0.67			
5				0.587			
6				0.688			
7					0.695		
8					0.68		
9					0.666		
10					0.539		
11					0.367		
12					0.386		
13	0.437						
14			0.555				
15			0.627				
16		0.617					
17		0.714					
18		0.74					
19		0.676					
20		0.71					
21		0.734					
22			0.529				
23			0.449				
24	0.589						
25	0.522						
26	0.574						
27	0.46						
28	0.451						
29			0.641				
30			0.679				
31							0.742
32							0.555
33							0.488
34						0.652	
35						0.739	
36						0.621	
37	0.714						
38	0.709						
39	0.577						
Eigenvalue	4.607	4.152	4.008	3.608	3.202	2.483	2.056
Variance factor %	11.813	10.647	10.276	9.251	8.211	6.367	5.273
Cumulative variance %	11.813	22.461	32.737	41.989	50.199	56.566	61.839
Name of factor	Support	Physical proximity	Accept and respect	Communi-cation	Warmth	Role sharing	Responsi-bility

**Table 4:** The factors and items of the well-being dimension

No item	Factor loading		
	F8	F9	F10
40			0.851
41			0.869
42			0.445
43		0.622	
44		0.843	
45		0.842	
46	0.663		
47		0.367	
48	0.681		
49	0.772		
50	0.794		
51	0.759		
52	0.817		
53	0.839		
54	0.793		
55	0.826		
56	0.496		
57	0.822		
Eigenvalue	6.587	2.669	2.584
Variance factor %	36.593	14.829	14.358
Cumulative variance %	36.593	51.422	65.78
Name of factor	Happiness	Problem-solving	Harmony

The factors formed from the EFA results were then analyzed using a CFA, and the data (440) were analyzed to test this construct. A CFA was performed using the maximum likelihood estimation. The theoretical construct consists of the dimensions of relationship quality and quality of well-being. The first dimension consisted of seven indicators: support, physical proximity, acceptance and respect, communication, warmth, sharing role, and responsibility. The dimension of well-being quality consists of three indicators: happiness, problem-solving, and harmony.

Various match indices were reported from the output analysis, and the suitability of the constructed model was determined using various criteria: RMSEA = 0.066 (< 0.08), SRMR = 0.055 (< 0.08), CFI = 0.99 (> 0.9), TLI = 0.99 (> 0.9), and NFI = 0.98 (> 0.9). Based on the criteria, it can be concluded that the data fit the Javanese marital quality model. All items had a significant estimator (*z* > 1.96 and *p* < 0.05).

The measurement of invariance multi-group has been analyzed from the fit model. There is a fit model from SRMR (< 0.08), CFI (> 0.9), TLI (> 0.9), and NFI (> 0.9). The model of measuring marital quality fits the group of husbands and wives. Each observed variable can also estimate the latent variable (*z* > 1.96 and *p* < 0.05). The difference in chi-square from the baseline, unconstrained, and constrained model was less than the chi-square table by 5%. The CFI multi-group difference is less than 0.01, indicating that Ho accepted. There are invariance models between groups or was not the difference in the measurement model in the two groups of husband and wife. Table 5 shows the fit and the calculation of the invariance models between groups.

**Table 5:** The invariance measurement model CFA between husband and wife

Label	*χ*2	df	CFI	TLI	NFI	SRMR	RMSEA
Baseline (b)	188	34	0,947	0.930	0.936	0.038	0.102
Unconstrain (un)	237	76	0,945	0.935	0.922	0.051	0.098
Constrain (c)	266	96	0,942	0.945	–	0.056	0.090
∆X^2^ un-c	29	∆df = 20	∆χ2 < χ2table 5%, Ho accepted				
∆X^2^ b-uc	49	∆df = 42	∆*χ*2<*χ*2table 5%, Ho accepted				
∆X^2^ b-c	78	∆df = 62	∆*χ*2<*χ*2table 5%, Ho accepted				
∆CFI uc-c	0,003	< 0.01	Ho accepted				
∆CFI b-uc	0,002	< 0.01	Ho accepted				
∆CFI b-c	0,005	< 0.01	Ho accepted				

### Convergent and discriminant analysis

The 420 dyadic data from the first and second studies were paired and analyzed to determine convergent and discriminant evidence. The correlation between the husband-and-wife factors can prove this validity, and the high correlation on the same factor between the husband-and-wife factors, compared to the correlation coefficient with other factors, demonstrated high evidence supporting validity. Likewise, the lower correlation coefficient for the correlation of different factors proved the discriminant of this measure. The coefficients of inter-dimensional correlation coefficients are presented in Table 6.

**Table 6:** The correlation coefficient between spouse factors

Husband	Wife									
	Sup	Phy	Accept	com	Warmth	Role	res	Happy	ps	Har
Sup	.643	.453	.512	.488	.484	.383	.417	.535	.369	.300
Phy	.463	.753	.530	.415	.421	.334	.328	.438	.313	.287
Accept	.636	.515	.703	.535	.543	.388	.454	.578	.454	.385
Com	.424	.315	.429	.581	.385	.217	.236	.320	.296	.220
warmth	.601	.406	.527	.578	.644	.351	.425	.564	.376	.279
Role	.537	.432	.490	.451	.433	.567	.436	.462	.375	.261
Res	.489	.388	.509	.385	.431	.445	.709	.420	.308	.301
Happy	.601	.469	.545	.515	.553	.416	.419	.690	.440	.348
Ps	.385	.335	.403	.357	.305	.286	.242	.368	.544	.342
Har	.311	.349	.387	.337	.253	.195	.283	.340	.372	.624

### Reliability estimation

Estimation reliability was analyzed on each dimension and composited for the whole instrument scale. The outcome of the reliability analysis of each dimension was using the alpha Cronbach formula and obtained the support result 0.91, physical proximity 0.86, acceptance and respect 0.83, communication 0.83, warmth 0.88, role sharing 0.81, responsibility 0.77, happiness 0.95, problem-solving 0.74, and harmony 0.76. Composite reliability is a method of measuring reliability based on the construct of the analysis variability in each dimension. Using the formula by McDonald (1999), the estimation of the reliability of relation quality was 0.901, and well-being was 0.853. The reliability estimation results in the study were good because they demonstrated each dimension with a reliability coefficient above 0.7. Based on these results, measurements of the marital quality of Javanese people’s instruments were consistent and trustworthy. These coefficients also point out the average extracted variant (AVE). The AVE relation quality was 0.567, and well-being was 0.659. These coefficients were at least 0.5, and this value indicates sufficient, which means that the latent variable can explain more than half of the indicators.

## Discussion and conclusion

The marital quality instrument in this study was built from instruments that Nurhayati has researched in some parts of Java (Nurhayati, 2017). By extending the study targets, psychometric analysis of property was carried out to obtain a model for measuring the quality of marriage for the Javanese, who make up the majority of the Indonesian population. Evidence of content validity was based on previous research as stated in the methodology. The items in this study were made from the results of the concept of marital quality which was built from qualitative research. The evident validity based on the content with Aiken analysis shows that all items are valid. Because this study examines more psychometric properties of a broader target and the items used are good and describe the quality of marriage, the content validity of this study still uses the evidence of validity from Nurhayati’s research.

**Fig. 1:**
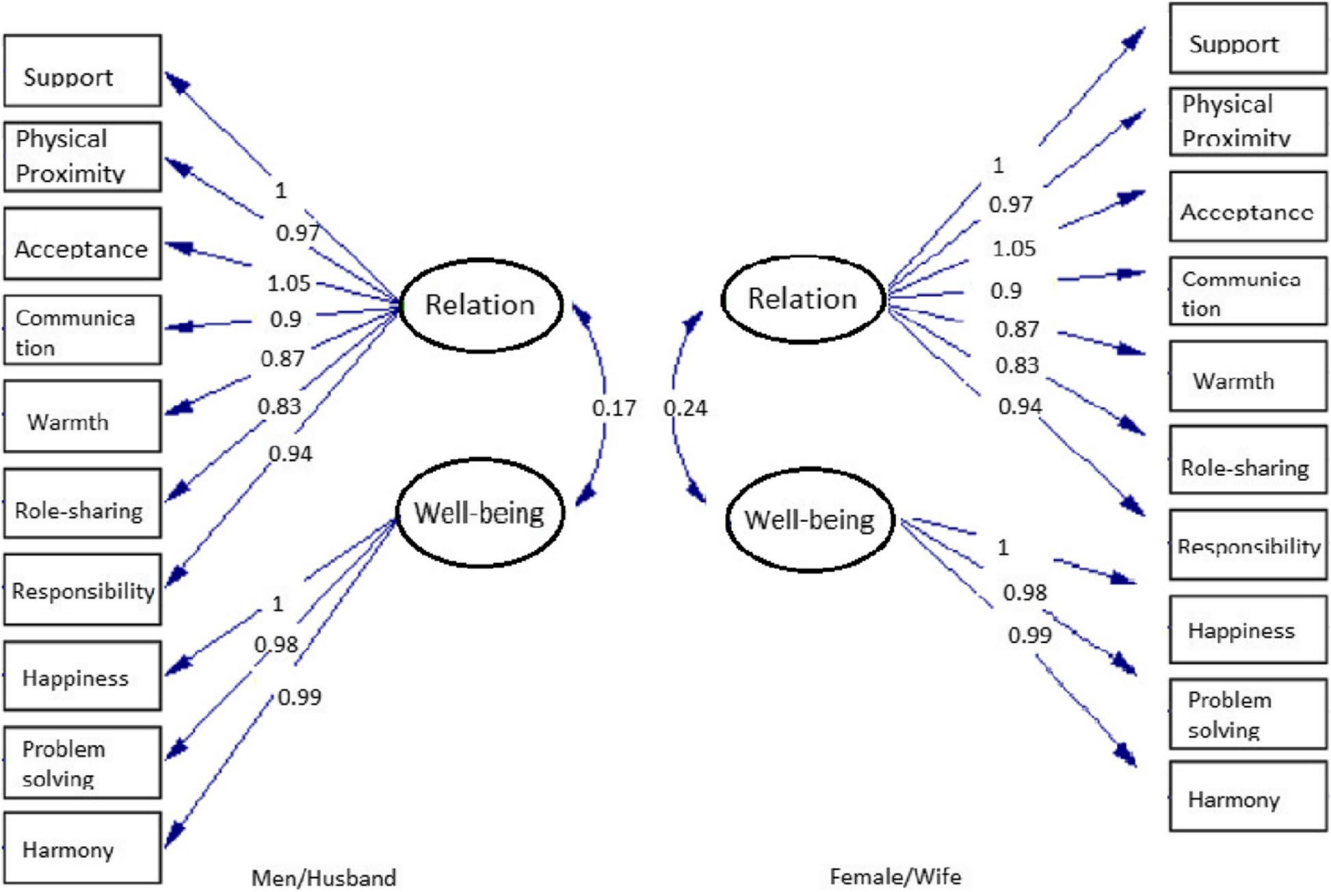
Measurement model of marital quality scale Javanese couples multi-group

The results of the EFA describe the construct of the Javanese marital quality scale and support the validity. Based on the data analysis, the Javanese marital quality measurement model was constructed (Fig. 1). This was demonstrated by the results of the first study, in which the construct of measurement model by the exploratory factor analysis demonstrated the dimension of relationship quality with a total contribution of 61.84% consisting of seven factors: support, physical proximity, acceptance and respect, communication, warmth, role-sharing, and responsibility. The well-being quality dimension had a total contribution of 65.78% and consisted of three factors: happiness, problem-solving, and harmony. The dimensions obtained from the EFA are slightly different from the initial concept built on the specifications of the instrument. Support, physical proximity, role-sharing, and responsibility and problem-solving are formed factors that previously did not become the name of the formed factors.

Marital quality instruments in this study were built from the uniqueness of Javanese marriages in Indonesia. The analysis results obtained the instrument’s psychometric properties, measured by construct instrument, convergent and discriminant analysis, and reliability. The construct of marital quality refers to the cultural concept (Munévar et al., 2021). The results of the construct validity analysis demonstrated that the Javanese marriage quality model could be built from the quality of relationships and the quality of well-being of husbands and wives, as obtained from several factors and the supporting items. The factor analysis identified factors that came from items grouped in the same term, and the CFA was used to test the construct of the measuring tool from the factors obtained by the EFA.

The CFA results obtained the fit measurement models on the marital quality construct. Relationship quality and well-being quality are the main components that shape marriage quality. The high quality of the relationship resulted in a high quality of well-being, and vice versa. Although the two dimensions were conceptually different in this model, relationship quality is a concept that involves other people, while the quality of well-being is an individual’s internal characteristics. Social relations are important for Javanese people, and this can be traced from expressions that indicate Javanese people’s social relations. Javanese people are known for the concepts of *guyub*, “harmony, friendly, cooperative,” and *sanak* “familiar relationship” (Sartono, 2010), demonstrating that they place great importance on harmony and social harmony (one component of harmony). The idiom *rukun agawe santosa kerah agawe bubrah*, or “harmony makes peace, the quarrel makes destruction,” a pillar of the order to make peace, demonstrates how Javanese people emphasize harmony. To maintain harmony in social relations, the Javanese people anticipate conflicts through teaching demonstrated by *wani ngalah luhur wekasane*, “want to give in, will get a high character,” or the person who concedes defeat will be nobler; another idiom state *sing uwis ya uwis:* “what happened was not necessary to be questioned.” These are the consequences for Javanese people’s marriages, which tend to value harmonious relations and avoidance of conflict. A harmonious couple demonstrates that they can manage family problems to minimize conflict, create individual responsibilities, and feel happy in the family. Therefore, harmony is one of the benchmarks of a good marriage (Ajrin, 2017; Nurhayati, 2017), and the Javanese will make various efforts to maintain harmony in their households.

Well-being in Javanese marriages cannot be separated from peace between the husband and wife (Ajrin, 2017), which in turn has a positive effect on happiness. Related to this feeling, the Javanese expression *kamulyaning urip dumunung ana tentreming ati*, “the glory of life is in the peace of heart,” demonstrates the importance of a sense of peace. This is in line with research considering well-being as the main goal of marriage (Fırat & Okanlı, 2019; Newsom et al., 2003) and relates to how husbands and wives build relationships (Amani & Khosroshahi, 2020). This indicates a strong association between relationship status and well-being in this research.

The results of the multi-group measurement model research show that there is no difference in the fit of the model for husbands and wives. Convergent and discriminant analysis shows a high correlation on the same dimensions between husbands and wives, compared to the relationship with other variables. Although the concept of Javanese marriage uses a patriarchal system, the results of this study show that the measurement model is suitable for groups of men and women, husband and wife both have the quality of marriage that can be seen in their relationship and well-being and their supporting aspects. The role of the wife, which began to shift by working in several public sectors, resulted in an equal relationship between husband and wife. A good relationship between husband and wife does not rule out the husband’s function as the head of the household and the wife’s function in managing the family. Good communication does not create the role of the husband must be higher than the wife. Good communication between husbands and wives will lead to a warm relationship (Fırat & Okanlı, 2019). Good communication, mutual acceptance, support, and collaborative problem-solving can bring about a happier and healthier family and an extended life (Liu & Waite, 2014; Robles, 2014; Slatcher & Schoebi, 2017; Zhang & Hayward, 2006).

This research instrument and the resulting information on psychometric properties can be helpful for researchers studying the marital quality of Javanese couples in particular. This study has several limitations. First, the validity evidence in this study focuses on the validity evidence based on the internal structure, convergent, discriminant. It has yet to include other instruments to support the external criteria. Subsequent studies can enhance the validity, analyzing the relationship with other tests in the same or different construct.

Second, likewise, although the results of the construct reliability demonstrate that this measuring instrument is quite reliable, the measurement for estimating reliability can be re-examined. Suppose further studies want to develop this measurement scale. In that case, those studies could use test-retest and alternate form reliability to complement Javanese quality marital psychometric property information. That also applied to the efforts to estimate the reliability method can be made by collecting the data again to obtain test-retest reliability.

Third, the results were determined using a self-report questionnaire, which allows for high social desirability but allows for dishonest responses. An explanation of the importance of answering honestly needs to be emphasized before working on this instrument so that respondents answer as they are. Consequently, the measurement results obtained will truly produce a true picture of the quality of marriage.

Fourth, a quality marital instrument was designed based on the concept of the marital in Javanese culture, which only consists of a husband-and-wife relationship. This instrument needs to be adjusted if it is to be used for couples with husband-wife characteristics that are not Javanese, including homosexual and heterosexual couples.

Although this study has some limitations, it has some practical implications. The instrument can be used to detect the quality of relationships and the well-being of husband and wife experiencing problems. The self-assessment instrument can be used by individuals to evaluate their marriage relationships. Furthermore, institutions dealing with divorce, such as the Department of Religion, can use the measurement for counseling or decision-making purposes for couples filing for divorce.

## Data Availability

The datasets used and/or analyzed during the current study are available from the corresponding author on reasonable request.

## Abbreviations

AERA: American Educational Research Association
APA: American Psychological Association
NCME: National Council on Measurement in Education
CFA: Confirmatory factor analysis
EFA: Exploratory factor analysis
KMO-MSA: Kaiser-Meyer-Olkin Measure of Sampling Adequacy
PMQ: Positive marital quality
NMQ: Negative marital quality
RMSEA: Root mean square error of approximation
SRMR: Standardized root mean square residual
CFI: Comparative fit index
TLI: Tucker-Lewis index
NFI: Bentler-Bonett Normed Fit Index
AVE: Average extracted variance
